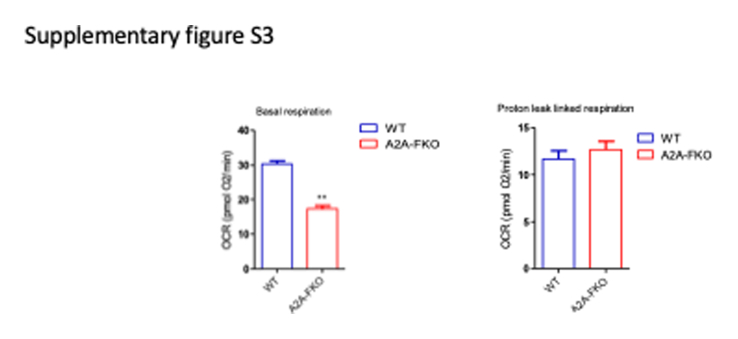# Metabolic dysfunction in mice with adipocyte-specific ablation of the adenosine A2A receptor

**DOI:** 10.1016/j.jbc.2025.108206

**Published:** 2025-01-17

**Authors:** Narendra Verma, Luce Perie, Michele Silvestro, Anupama Verma, Bruce N. Cronstein, Bhama Ramkhelawon, Elisabetta Mueller

**Affiliations:** 1 Holman Division of Endocrinology, Diabetes and Metabolism, Department of Medicine, New York University Grossman School of Medicine, New York, New York, USA; 2 Department of Systems Biology, Center of Biomedical Research, SGPGI campus, Lucknow, India; 3 Division of Vascular and Endovascular Surgery, Department of Surgery, New York University Grossman School of Medicine, New York, New York, USA; 4 Division of Translational Medicine, Department of Medicine, New York University Grossman School of Medicine, New York, New York, USA

**Keywords:** obesity, insulin resistance, brown and beige adipose tissue, adenosine A2A receptor, UCP1

## Abstract

It has been well established that adenosine plays a key role in the control of inflammation through G protein coupled receptors and recently shown that it can regulate thermogenesis. Here we investigated the specific requirements of the adenosine A2A receptor (A2AR) in mature adipocytes for thermogenic functionality and metabolic homeostasis. We generated fat tissue-specific adenosine A2AR KO mice to assess the influence of signaling through this receptor on brown and beige fat functionality, obesity, insulin sensitivity, inflammation, and liver function. Fat-specific A2AR KO and WT littermate mice were compared for potential differences in cold tolerance and energy metabolism. In addition, we measured glucose metabolism, AT inflammation, and liver phenotypes in mice of the two genotypes after exposure to a diet rich in fat. Our results provide novel evidence indicating that loss of the adenosine A2AR specifically in adipocytes is associated with cold intolerance and decreased oxygen consumption. Furthermore, mice with fat specific ablation of the A2AR exposed to a diet rich in fat showed increased propensity to obesity, decreased insulin sensitivity, elevated adipose tissue inflammation, and hepato-steatosis and hepato-steatitis. Overall, our data provide novel evidence that A2AR in mature adipocytes safeguards metabolic homeostasis, suggesting the possibility of targeting this receptor selectively in fat for the treatment of metabolic disease.

Brown and beige fat tissues are key endocrine organs involved in the regulation of glucose and lipid homeostasis. We, and others, have previously demonstrated that activation of brown/beige adipocytes can improve diabetes (1, 2, 3), suggesting the possibility of targeting brown/beige tissues to combat obesity and circumvent its associated morbidities. One of the central regulators of beige and brown adipose tissue functionality is the uncoupling protein 1 (UCP1), a protein involved in the uncoupling of respiration from the generation of energy in the form of ATP, leading to proton leak and thermogenesis (4). In addition to UCP1, factors such as Cidea, Dio2, HSF1, and ATGL regulate brown and beige fat functionality and are highly induced upon cold stimuli or *via* pharmacological activation of β-adrenergic signaling (2, 3, 5, 6, 7, 8).

Adenosine is a purine nucleoside derived from the degradation of ATP, ADP, and AMP, which signals through ligation to the purinergic receptors A1, A2A, A2B, and A3 (9). It has been demonstrated that these types of G protein–coupled receptors are differentially expressed in tissues (10). Specifically, it has been shown that the adenosine A2A receptor is present at high levels in both beige and brown fat adipocytes and that whole-body deletion of A2AR in mice is accompanied by reduced brown adipose tissue (BAT) activation and diminished nonshivering thermogenesis (11). Moreover, systemic activation of the A2AR by specific agonists in obese mice leads to decreased white adipose tissue (WAT) expansion and causes weight loss (11). More recently, our studies have demonstrated that pharmacological increase in adenosine levels *in vivo* in obese mice leads to decrease in body weight and reduces inflammation (12). Despite these published data (11, 13, 14), the direct effects of A2AR signaling selectively in fat tissue remain undefined. Here we provide novel evidence demonstrating that mice specifically lacking the A2AR in mature adipocytes have reduced expression of thermogenic genes in adipose tissue and decreased thermogenesis. Furthermore, we show that fat-specific A2AR KO mice (from now on referred to as A2AR-FKO mice) exposed to a high fat diet (HFD) have decreased insulin sensitivity and elevated inflammation in visceral adipose tissue. Analysis of livers obtained from A2AR-FKO mice revealed increased hepato-steatosis and hepato-steatitis. Overall, our studies demonstrate that loss of A2AR specifically in fat cells alters brown adipose tissue functionality and increases metabolic dysfunction in response to high caloric intake, providing novel insights into the mechanisms that regulate adipose tissue functionality and uncovering a potential new tissue-specific therapeutic target to combat obesity and metabolic dysfunction.

## Results

### Generation of adenosine A2A receptor adipocyte-specific KO mice

**Figure 1:**
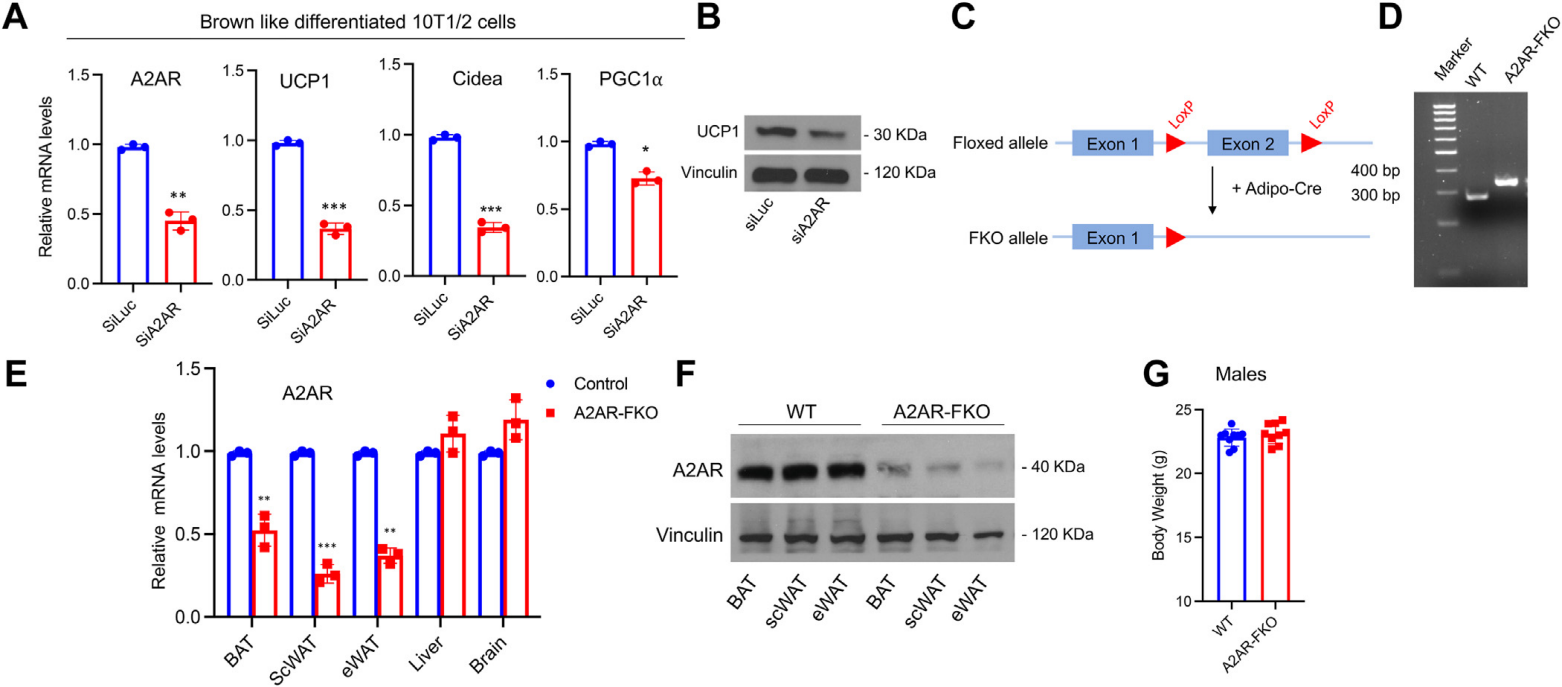
**Fat cell autonomous induction of browning requires the adenosine A2A receptor and generation of adipose tissue–specific A2AR KO (A2AR-FKO) mice**. *A*, A2AR, UCP1, Cidea, and PGC1α relative mRNA levels in brown-like differentiated 10T1/2 cells transfected with either siLuc or siA2AR. *B*, Western blot analysis of UCP1 levels in brown-like differentiated 10T1/2 cells transfected with either siLuc or siA2AR. *C*, schematic representation of the strategy used to generate tissue-specific A2AR-FKO mice. *D*, genotyping analysis of control and A2AR-FKO mice. *E*, relative mRNA levels of A2AR in tissues of control and A2AR-FKO mice (n = 3). *F*, Western blot analysis of A2AR levels in control and A2AR-FKO mice. *G*, total body weight of 10-week-old male (n = 9) mice on a normal chow diet. Results are expressed as a mean ± SD from three independent experiments and ∗ *p* value < 0.05; ∗∗*p* value < 0.005; ∗∗∗*p* value < 0.001.

**Figure S1:**
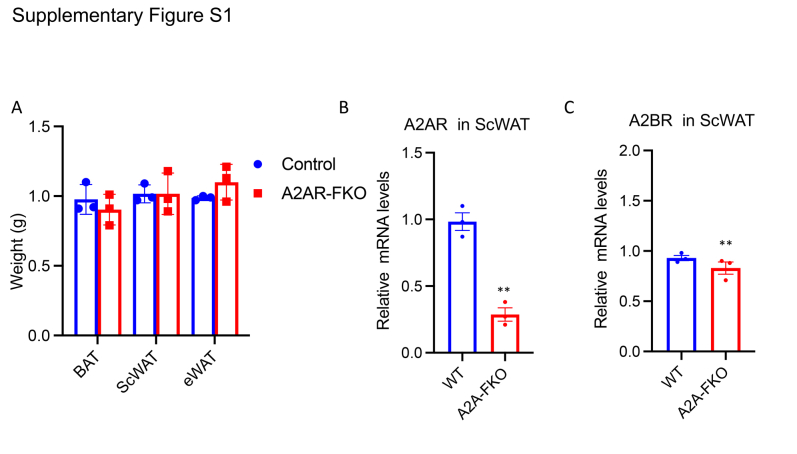


Although it has been shown that adenosine signaling regulates brown fat functionality (11), it has not yet been assessed whether the adenosine A2A receptor (A2AR) present in adipocytes plays a specific role in this process and in the regulation of metabolism. To first determine whether signaling through the A2AR present in mature fat cells is directly involved in the regulation of brown fat genes, we first performed loss-of-function experiments in the multipotent 10T1/2 cells that can be induced to differentiate into adipocytes *in vitro* (6). Knock-down of A2AR by siRNA in brown-like differentiated 10T1/2 adipocytes led to a decrease in the mRNA levels of classic beige/brown adipocyte markers such as UCP1, PGC1α, and Cidea mRNAs, compared to adipocytes transfected with control silucRNA (Fig. 1*A*). Western blot analysis confirmed that the decrease in UCP1 occurs also at the protein level (Fig. 1*B*). To assess *in vivo* the influence of the adenosine A2AR on brown fat functionality, we generated fat-specific A2AR KO mice by crossing A2AR-loxP mice with Adiponectin-Cre mice (Fig. 1, *C* and *D*). To confirm the specific ablation of A2AR in fat, we analyzed A2AR levels in various tissues and demonstrated loss of A2AR in an adipose tissue–selective manner in A2AR KO mice (Fig. 1, *E* and *F*). The reduction in A2AR levels did not lead to a compensatory upregulation of the A2B receptor, as shown in Fig. S1. Initial phenotypical analysis of WT and adipose-selective A2AR KO mice at 10 weeks of age fed a normal diet demonstrated that the two genotypes have comparable body weight (Fig. 1*G*).

### Loss of A2AR in fat decreases cold tolerance

**Figure 2:**
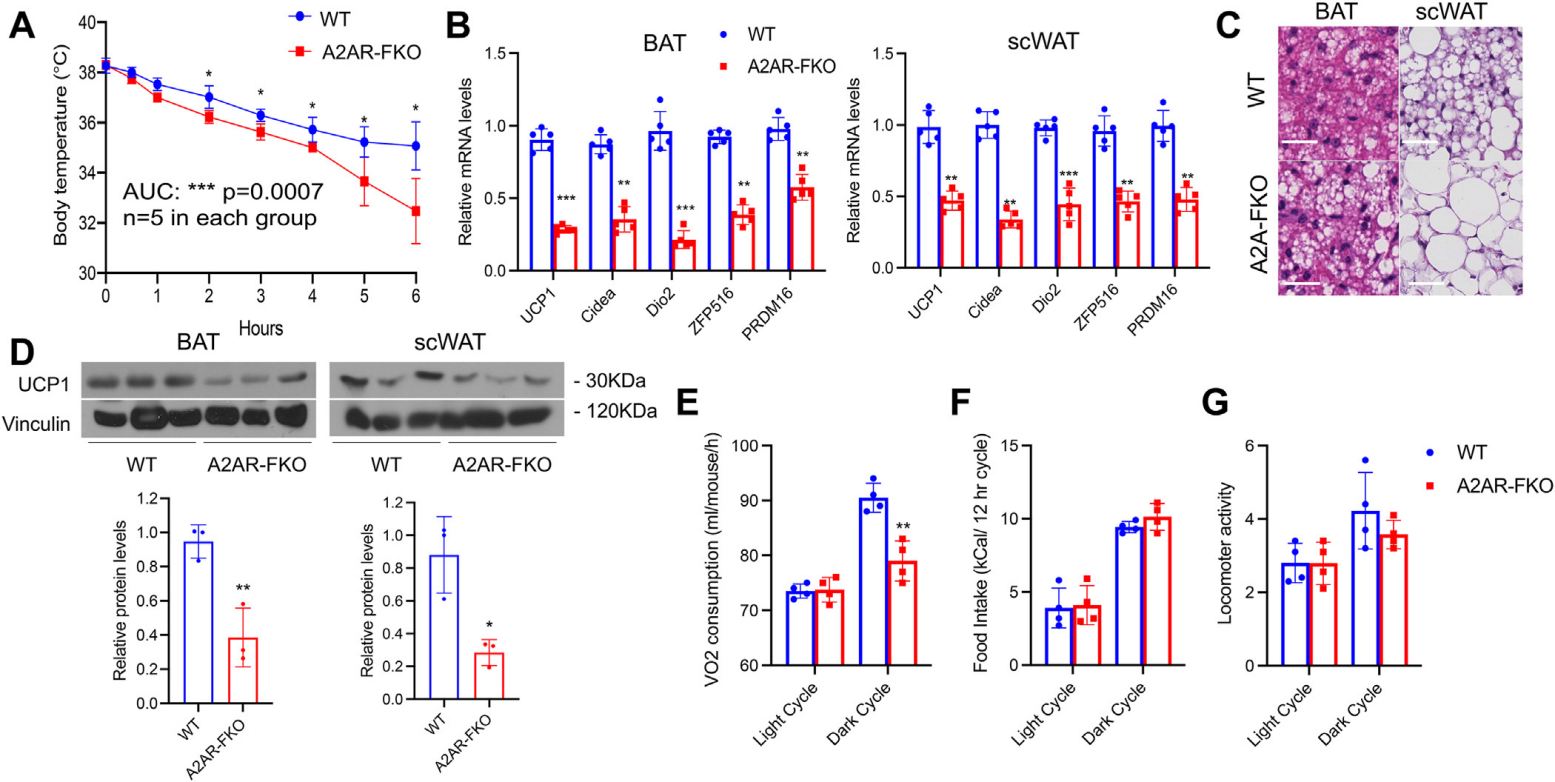
**Specific loss of A2AR in adipocytes reduces cold resistance and decreases oxygen consumption**. *A*, core temperatures in control and A2AR-FKO male mice at 10 weeks of age exposed to 4 °C. n = 5. *B*, relative mRNA levels of UCP1, Cidea, Dio2, ZFP516, and PRDM16 in BAT and scWAT of control and A2AR-FKO mice at the end of the cold exposure experiment. *C*, H & E staining of BAT and scWAT of control and A2AR-FKO mice. Magnification, ×40; scale bar represents 100 μm. *D*, Western blot analysis of UCP1 levels in scWAT and BAT of control and A2AR-FKO mice and quantification of protein levels. Vinculin was used as a loading control. *E*, oxygen consumption in control and A2AR-FKO mice (n = 4). *F*, food intake in control and A2AR-FKO mice. n = 4. *G*, locomotor activity in control and A2AR-FKO mice (n = 4). Results are expressed as a mean ± SD and ∗ *p* value < 0.05; ∗∗*p* value < 0.005; ∗∗∗*p* value < 0.001.

**Figure 3:**
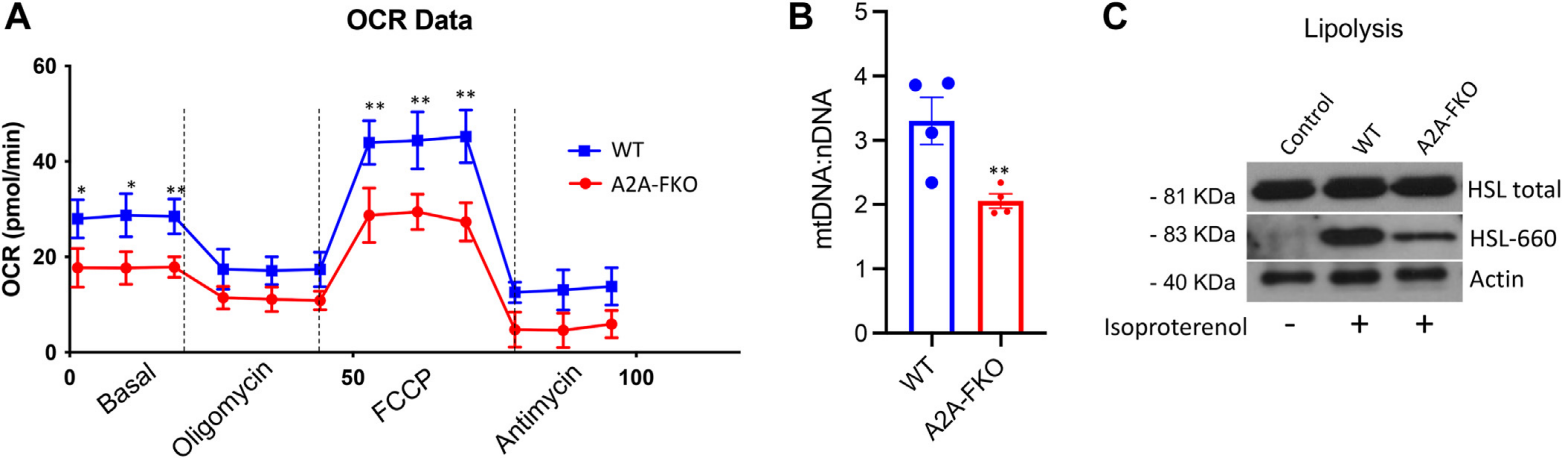
**Cells lacking A2AR have intrinsic defects in respiration and lipolysis**. *A*, seahorse analysis of brown-like adipocytes differentiated from SVF cells obtained from the scWAT of control and A2AR-FKO mice. *B*, ratio of mitochondrial DNA and nuclear DNA in brown-like adipocytes differentiated from SVF cells obtained from the scWAT of control and A2AR-FKO mice. *C*, Western blot analysis of protein levels of HSL and phospho-HSL in brown-like differentiated adipocytes obtained from SVF cells of control and A2AR-FKO mice treated with isoproterenol. Actin was used a loading control. Results are expressed as a mean ± SD and ∗ *p* value < 0.05; ∗∗*p* value < 0.005; ∗∗∗*p* value < 0.001.

Given the evidence that knock-down of the A2AR in differentiated 10T1/2 adipocytes reduced the levels of brown fat markers *in vitro*, we tested whether loss of adenosine signaling through A2AR selectively in fat could alter thermogenic responses. To test this, we exposed 10-week-old control and A2AR-FKO male mice to cold temperatures (4 °C) for 6 h. This analysis revealed that loss of A2AR in adipocytes is associated with a reduction in cold tolerance (Fig. 2*A*). To determine whether the defective thermogenic response observed in A2AR-FKO mice was accompanied by a reduction in the induction of thermogenic genes, we performed molecular analysis of fat tissues biopsies obtained from the mice of both genotypes. mRNA studies revealed that fat tissues of A2AR-FKO mice exposed to cold have decreased levels of UCP1, Cidea, and Dio2 and of genes, such as ZFP516 and PRDM16 (Fig. 2*B*), previously known to be regulated in response to cold exposure in thermogenic tissues (2, 6, 15, 16, 17). H & E staining of BAT and scWAT demonstrated increased lipid accumulation in the BAT and scWAT of A2AR-FKO mice compared to control (Fig. 2*C*), and Western blot analysis confirmed the decrease in UCP1 also at the protein levels (Fig. 2*D*). Altogether, these data demonstrate that A2AR-FKO mice have reduced thermogenic gene expression programs and UCP1 levels, increased lipid deposits in thermogenic tissues *in vivo*, and decreased cold resistance. To assess whether energy expenditure is differentially regulated in control and A2AR-FKO mice, we placed control and A2AR-FKO mice in metabolic cages. As shown in Figure 2*E*, mice with fat-specific loss of A2AR showed reduced oxygen consumption, while their food intake (Fig. 2*F*) and locomotor activity (Fig. 2*G*) were comparable to their littermate control mice. To assess whether the differences observed were due to cell-autonomous abnormalities, we measured several parameters in isolated primary adipocytes obtained from subcutaneous fat tissue of control and A2AR-FKO mice. Specifically, Seahorse analysis revealed reduced oxygen consumption (Figs. 3*A* and S3) and measurements of mitochondrial DNA showed decreased mitochondria amount in A2AR-FKO adipocytes (Fig. 3*B*) compared to control cells. Analysis of HSL levels demonstrated reduced phosphorylation at serine 660, indicating decreased lipolytic activity in adipocytes lacking the A2AR (Fig. 3*C*).

### Increased propensity to develop obesity after HFD in mice with loss of A2AR specifically in fat tissue

**Figure 4:**
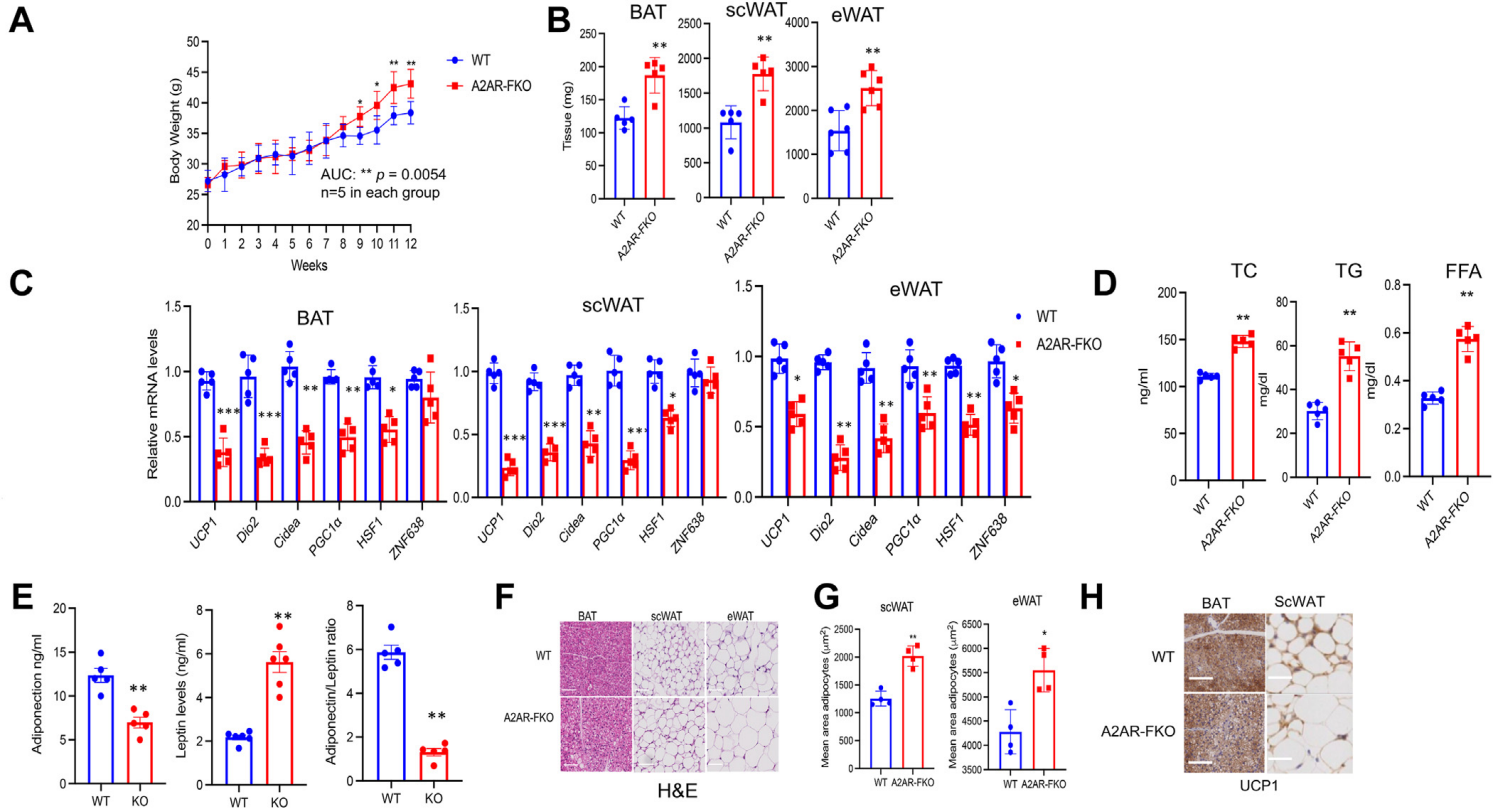
**A2AR-FKO mice have increased propensity to obesity after HFD**. *A*, total body weight over time, (*B*) adipose tissues weight, and (*C*) relative mRNA levels of UCP1, Dio2, Cidea, PGC1α, HSF1, and ZNF638 in the BAT, scWAT, and eWAT of mice treated for 12 weeks with HFD (n = 5). *D*, serum parameters in control and A2AR-FKO mice (n = 5). *E*, adiponectin and leptin levels and their ratio in control and A2AR-FKO mice (KO). *F*, H&E staining of fat depots of control and A2AR-FKO mice. Magnification, ×40; scale bar represents 100 μm. *G*, quantification of adipocyte size in scWAT and eWAT of control and A2AR-FKO. *H*, UCP1 immunohistochemistry in the BAT and scWAT of control and A2AR-FKO mice. Results are expressed as a mean ± SD and ∗ *p* value < 0.05; ∗∗*p* value < 0.005; ∗∗∗*p* value < 0.001.

Given the reduced oxygen consumption we observed in primary cells and *in vivo*, in mice lacking A2AR in fat on a normal diet, and the decreased lipolysis in cells lacking the A2AR, we tested whether nutritional overload would increase the propensity of A2AR-FKO to develop obesity. Analysis of control and A2AR-FKO male mice fed a HFD for 12 weeks demonstrated that loss of A2AR in fat is associated with increased body weight (Fig. 4*A*). Further detailed analyses revealed increased amounts of adipose depots in A2AR-FKO compared to control mice (Fig. 4*B*) and decreased expression of thermogenic markers (Fig. 4*C*) in all three depots of A2AR-FKO mice. Assessment of serum parameters indicated significant increase in total cholesterol levels, triglyceride, and free fatty acids (Fig. 4*D*) and reduced levels of the adipokines leptin and adiponectin (Fig. 4*E*) in A2AR-FKO mice. Histological analysis *via* H&E staining (Fig. 4*F*) showed morphological changes demonstrating an increase in the size of the adipocytes of fat depots of A2AR-FKO mice, compared to control. These data, confirmed by quantitative analysis of the adipocytes area (Fig. 4*G*), demonstrate increased lipid deposition in the absence of A2AR in fat. Furthermore, immunohistochemical analysis of BAT and scWAT revealed decreased UCP1 staining in A2AR-FKO mice (Fig. 4*H*).

### Decreased insulin sensitivity and elevated AT inflammation in A2AR-FKO mice

**Figure 5:**
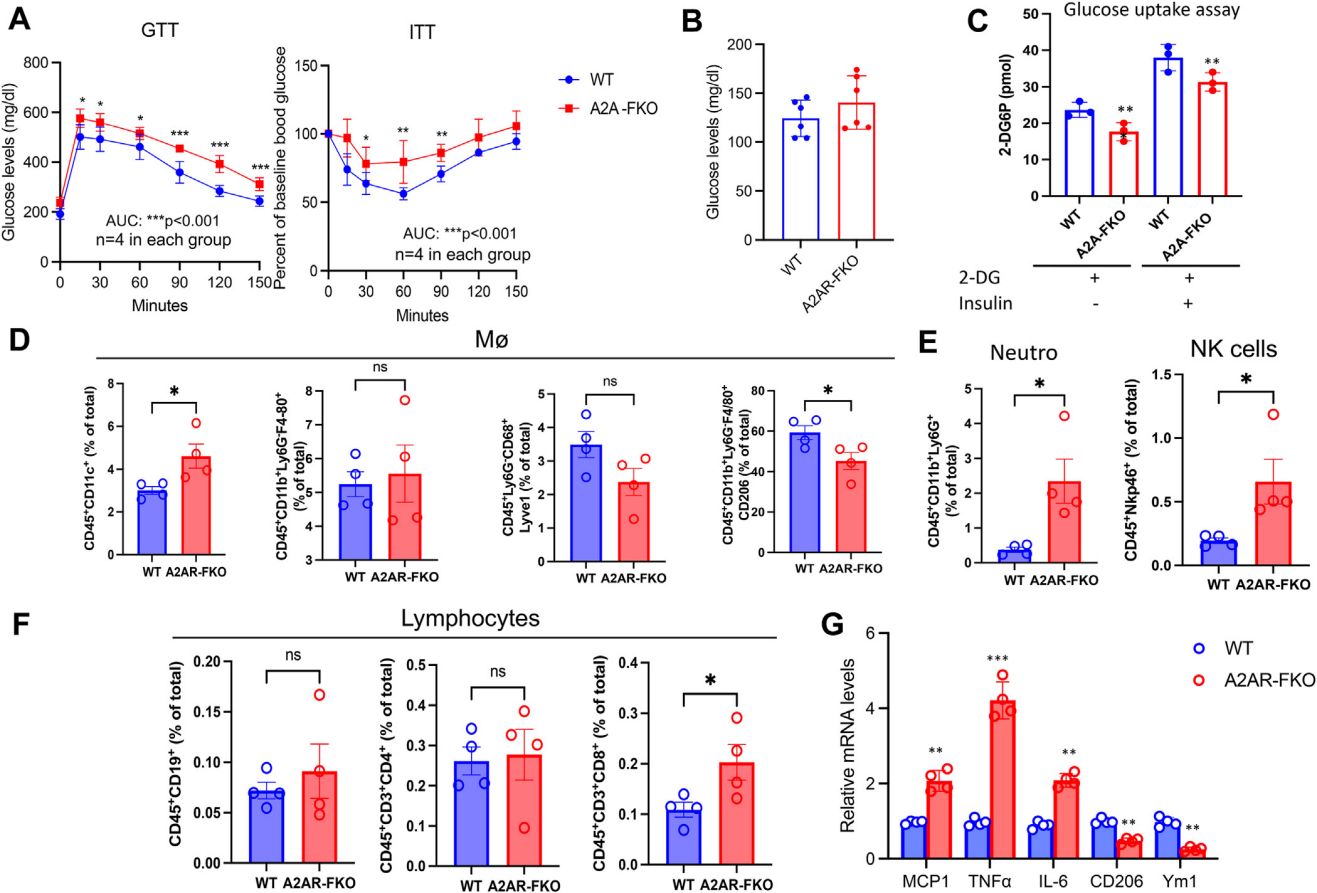
**Decreased insulin sensitivity and increased inflammation in A2AR-FKO mice**. *A*, GTT and ITT assays and (*B*) basal glucose levels of control and A2AR-FKO mice treated with HFD for 12 weeks. *C*, glucose uptake assay in brown-like differentiated SVF cells derived from the scWAT of control and A2AR-KO mice. *D*–*F*, flow cytometry analysis of inflammatory cells obtained from the eWAT of control and A2AR-FKO mice on a HFD for 12 weeks. *G*, relative mRNA levels of genes related to inflammation in the eWAT of control and A2AR-FKO mice exposed to HFD for 12 weeks. Experiments were performed in male mice, n = 4 per group. Results are expressed as a mean ± SD and ∗ *p* value < 0.05; ∗∗*p* value < 0.005; ∗∗∗*p* value < 0.001. DC, dendritic cells; Mø, macrophages.

Given the evidence that we, and others, have provided that activation of brown fat function is associated with improved glucose homeostasis (1, 18), we determined the effects of loss of adenosine signaling through A2AR specifically in fat on overall metabolic dysfunction associated with nutritional overload. We first assessed whether loss of A2AR in fat is accompanied by changes in glucose metabolism. Our analysis revealed that A2AR-FKO mice have decreased insulin sensitivity, as shown by GTT and ITT assays (Fig. 5*A*) while chow-fed control and A2AR-FKO mice had comparable baseline glucose levels (Fig. 5*B*). To assess whether the effects on glucose metabolism were intrinsic to cells lacking the A2AR, we performed analysis of glucose uptake in control and A2AR KO–isolated adipocytes. Our results demonstrate a cell-autonomous impairment in this process in A2AR-KO cells, as shown in Figure 5*C*.

**Figure S2:**
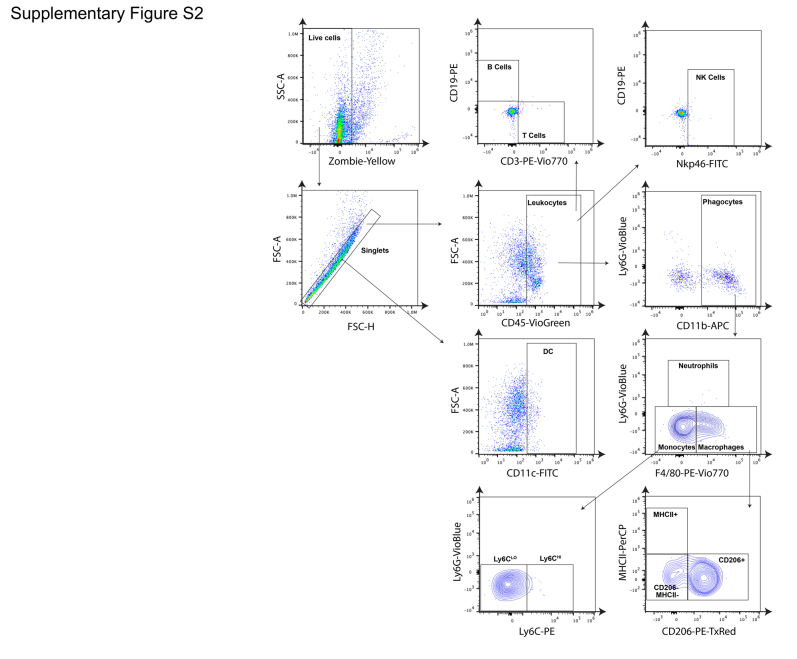


Further analysis of the visceral fat depot by flow cytometry (Fig. S2) revealed an increase of CD11C^+^ subsets (Fig. 5*D*). F4/80^+^ macrophages were not significantly different between the two genotypes, while we observed a trend in decreased accumulation of resident Lyve1^+^ macrophages (Fig. 5*D*). Furthermore, we observed a decrease in CD206^+^ macrophages (Fig. 5*D*), consistently with an elevated inflammatory adipose milieu and increased expression of neutrophils and of NK cells (Fig. 5*E*) in eWAT of A2AR-FKO. While no differences in the levels of B and CD4^+^ lymphocytes were noted between the two genotypes (Fig. 5*F*), we observed a surge in CD8^+^ lymphocytes in the fat of A2AR-FKO mice. These data suggest a dysregulated distribution of immune response in fat during high fat feeding in mice with selective ablation of A2AR in adipocytes. Molecular analysis of the expression of a panel of genes encoding for proteins involved in inflammation demonstrated increased expression of MCP1, TNFα, and IL-6 and decreased levels of CD206 and Ym1 (Fig. 5*G*) in the eWAT of A2AR-FKO mice. These data demonstrate that loss of A2AR specifically in fat increases inflammation and reduces M2 polarization in white adipose tissue. Altogether, our results suggest that the A2AR present in adipocytes is required to maintain insulin sensitivity and to reduce inflammation in adipose tissue.

### Adipocyte-specific loss of the adenosine A2AR causes fatty liver and hepato-steatitis

**Figure 6:**
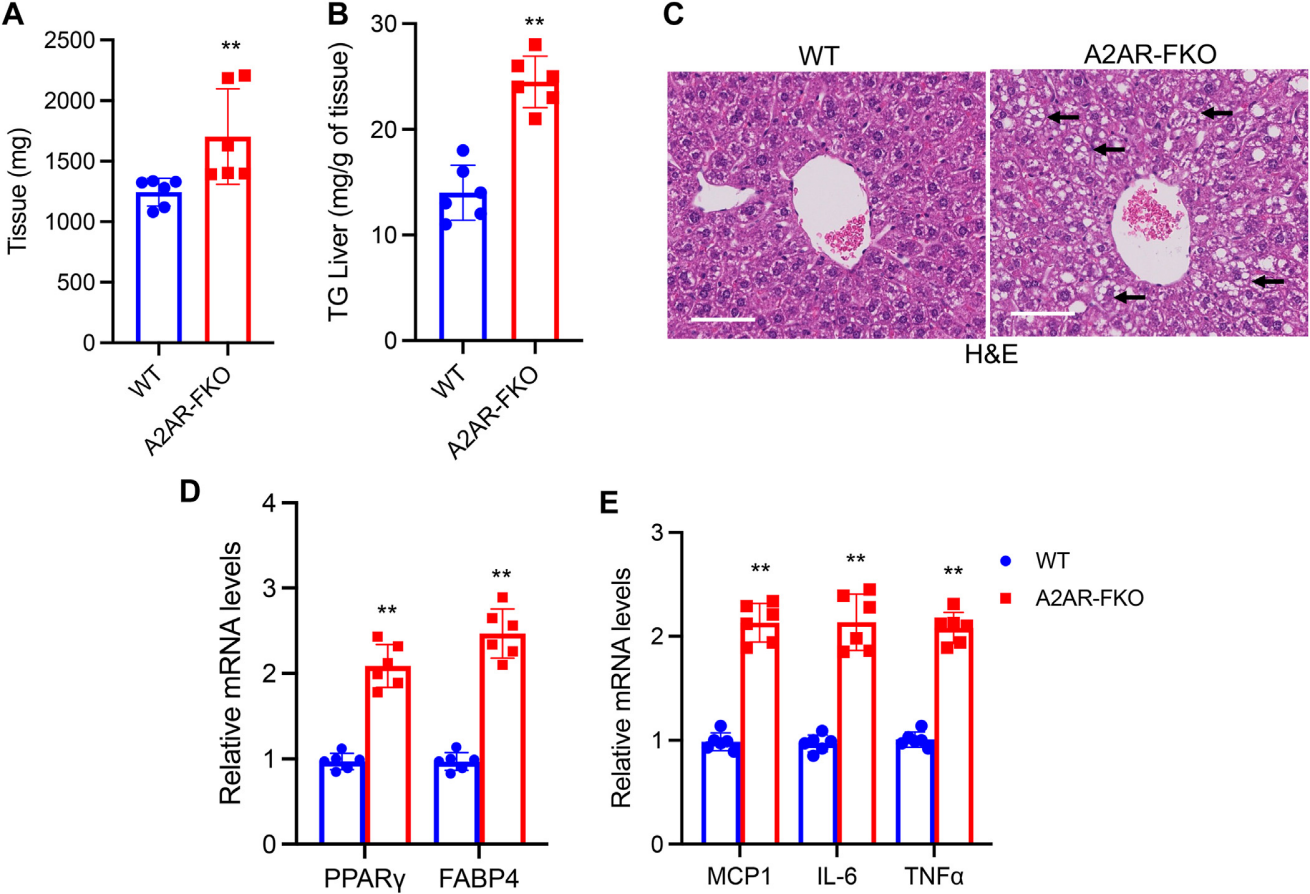
**Development of fatty liver and hepatic steatitis in A2AR-FKO mice after HFD**. *A*, Liver tissue weight and (*B*) TG levels of control and A2AR-FKO mice after exposure to a HFD for 12 weeks (n = 6). *C*, H&E staining in control and A2AR-FKO mice after exposure to a HFD for 12 weeks. Magnification, ×40; scale bar represents 100 μm. *Black arrows* point to multilocular cells with lipids. *D*, relative mRNA levels of PPARγ and FABP4 in liver biopsies obtained from control and A2AR-FKO mice after 12 weeks on HFD. *E*, relative mRNA levels of pro-inflammatory genes in the liver biopsies of control and A2AR-FKO mice after 12 weeks on HFD. *Black* arrows point to multilocular lipid deposits. Results are expressed as a mean ± SD and ∗ *p* value < 0.05; ∗∗*p* value < 0.005; ∗∗∗*p* value < 0.001.

**Figure 7:**
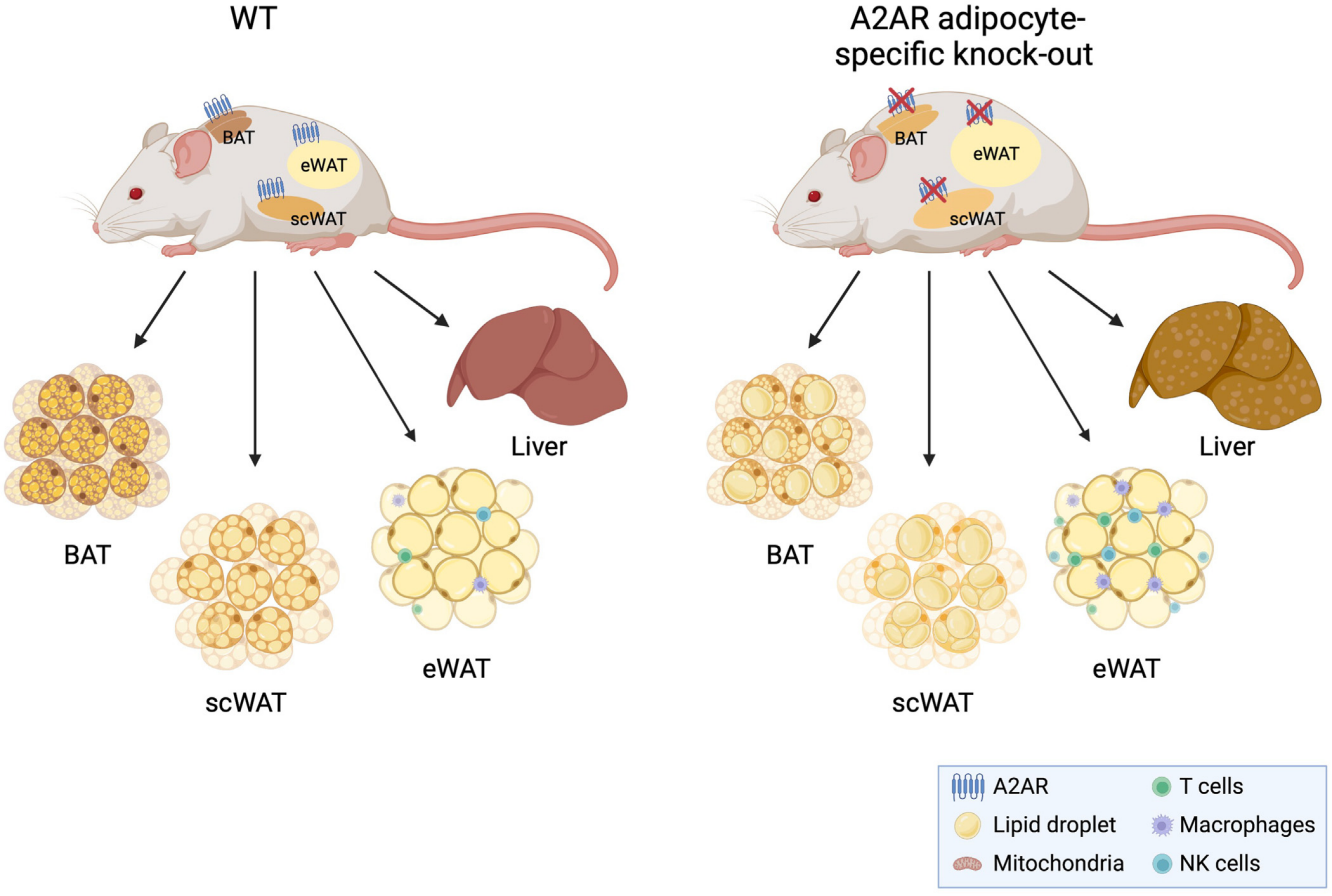
**Schematic representation of the specific role of adenosine A2A receptor in fat in the maintenance of metabolic homeostasis**. Loss of adenosine A2A receptor specifically in fat is accompanied by increased lipid accumulation, decreased browning of fat tissues, increased overall obesity, elevated adipose tissue inflammation, and hepatic steatosis and steatitis.

Obese states often lead to hepato-steatosis due to ectopic lipid accumulation. In order to determine the effects of HFD in control and A2AR-FKO mice, we performed gross pathology, in addition to histological and molecular analysis, of livers samples. Tissue measurements revealed that loss of A2AR in adipose tissue leads to a significant increase in liver weight in A2AR-FKO mice, compared to control control mice (Fig. 6*A*). Furthermore, triglyceride analysis revealed increased levels in A2AR-FKO mice (Fig. 6*B*) and H&E staining showed increased lipid accumulation (Fig. 6*C*). Molecular studies revealed altered expression of genes contributing to lipid deposition, such as PPARγ and FABP4 (Fig. 6*D*), in the livers of A2AR-FKO mice, compared to control littermate mice, and increased levels of genes encoding for proteins involved in inflammation, such as MCP1, IL6, and TNFα (Fig. 6*E*). Altogether, our data demonstrate that loss of adenosine signaling through the A2AR in fat affects obesity, glucose metabolism, and contributes to fatty liver disease and steatitis (Fig. 7).

## Discussion

Adenosine has been previously shown to counteract inflammation in various organs through select G protein–coupled receptor signaling pathways, providing a strong rationale for the development of adenosine-like agonists for the treatment of inflammatory and autoimmune disorders (19, 20, 21). More recently, it has been shown that interfering with adenosine signaling through global disruption of the A2AR (11, 22) or *via* deletion of A2B receptor specifically in fat (23) can alter thermogenesis, suggesting a critical role of these receptors in metabolism. Specifically, it has been shown that pharmacological activation of the A2B receptor maintains metabolic health during the aging process through its combined and coordinated action on skeletal muscle and brown fat. Despite the improved understanding of the role of specific G protein–coupled receptors in energy expenditure and heat generation, little is known about their absolute requirements selectively in adipose tissue to maintain overall metabolic homeostasis.

To define the function of the A2AR in mature adipocytes *in vivo* and to determine whether fat-specific A2AR is necessary to prevent obesity and related metabolic dysfunction in the conditions of caloric overload, we generated conditional KO mice with the ablation of A2AR specifically in fat depots (A2AR-FKO). Our data demonstrate that A2AR-FKO mice have decreased thermogenesis associated with reduced brown fat gene expression in adipose tissues. These defects are fat cell-autonomous, given that markers of brown fat function are reduced in *in vitro*–differentiated cells with knock-down of A2AR. Furthermore, our analysis of A2AR-FKO mice on a normal diet has evidenced decreased oxygen consumption compared to control mice, despite similar food intake and locomotor activity. These data support the thermogenesis defects observed in A2AR-FKO mice. Analysis of isolated adipocytes derived from control and A2AR-FKO mice show that oxygen consumption and mitochondrial amount are decreased in the absence of the A2AR, demonstrating that the thermogenic defects are cell autonomous. Given that previous reports (11, 22, 24) have not determined the requirements of A2AR selectively in fat tissue and cells for the regulation of oxygen consumption, we believe that our data are the first to indicate the effects of loss of A2AR in fat tissue on energy balance and thermogenic responses.

The results we have obtained after challenging mice with nutritional overload indicate increased propensity of A2AR-FKO mice to develop obesity, insulin resistance, liver steatosis, and steatitis, compared to control littermate mice. These data suggest that A2AR function in adipose tissues contributes to the maintenance of metabolic homeostasis. Although previous reports have shown that mice with global deletion of the A2AR have impaired thermogenic responses (11), the question of whether dysfunction of A2AR signaling has direct or indirect effects on adipose tissue biology and functionality has not been addressed. Our results demonstrate that mice with adipocyte-specific loss of A2AR-FKO have a significant increase in overall body weight and in adipose tissue amounts. These changes in A2AR-FKO mice are accompanied by decreased levels of expression of thermogenic genes in adipose tissues, further supporting the observation of decreased energy expenditure observed in the KO mice, associated with increased lipid deposition and adipose depots expansion. Given that increase in fat mass is generally accompanied by dysfunction in glucose homeostasis, we challenged the mice with GTT and ITT assays and demonstrated that A2AR-FKO mice exposed to HFD have impaired insulin sensitivity. These effects could be secondary due to the overall increase in body weight, given that insulin sensitivity is generally affected in many animal models of obesity. Our data obtained in isolated adipocytes strongly suggest that the decreased insulin sensitivity of A2AR-FKO mice is due to a cell autonomous defect, as shown by the decrease glucose uptake in cultured adipocytes lacking A2AR. These data provide evidence that adipocytes lacking the A2AR have an intrinsic impairment in glucose uptake. Through further molecular and histological characterization, we show that increased fat deposition in the eWAT of A2AR-FKO mice is associated with elevated M1 polarization, increased expression of inflammatory markers, and dysregulated immune distribution. Our detailed flow cytometry analysis also revealed an increase in the proportion of NK cells in A2AR-FKO mice compared to control, which appears to be consistent with the phenotype previously reported in mice with global loss of A2AR signaling (25). These data suggest that loss of A2AR signaling specifically in fat may promote natural killer cell maturation and supports the notion that adenosine signaling through A2AR regulates the cross-talk between adipocytes and immune cells. Overall, these data show that loss of A2AR signaling in fat tissues leads to decreased insulin sensitivity and increased inflammation, demonstrating that A2AR signaling in adipocytes has an impact on glucose homeostasis and overall AT inflammation. The increased presence of neutrophils in the mutant mice suggests an exacerbated inflammatory state, which aligns with prior evidence linking chronic low-grade inflammation in obesity with elevated neutrophil infiltration (26, 27). This could indicate that the selective ablation of A2AR in adipocytes alters immune cell trafficking or retention, contributing to a dysregulated immune response during HFD exposure. The increased neutrophil infiltration may also reflect impaired resolution of inflammation in the absence of A2AR signaling. A2AR is known to exert anti-inflammatory signaling (19, 20, 21), and its deletion in adipocytes could result in enhanced recruitment or delayed clearance of neutrophils, fueling inflammation. Our findings support the concept that A2AR signaling plays a role in immune homeostasis in adipose tissue under metabolic stress, and its disruption can lead to an imbalanced immune response.

Our data reveal that loss of A2AR specifically in fat in conditions of nutritional overload leads to increased liver mass. These effects are accompanied by elevated hepatic lipid deposition in A2AR-FKO mice in comparison to control littermates, as shown by histological and molecular analyses. Specifically, A2AR-FKO mice exposed to HFD display lipotoxicity, as demonstrated by the increase in hepatic triglycerides accumulation and in the elevation of the expression of genes involved in fatty liver disease and inflammation. All together, our results support the evidence that loss of A2AR in adipocytes is associated with hepatic steatosis and steatitis in conditions of nutritional overload. These findings are new since no reports to date have shown the necessity of intact A2AR signaling in fat for the maintenance of liver functionality. Altogether, our data demonstrate for the first time the requirement of A2AR in fat tissues in the regulation of metabolic homeostasis and suggest the possibility of developing fat tissue–selective A2AR agonists for therapeutic use to combat obesity and associated metabolic dysfunction.

## Experimental procedures

### Cell culture and treatments

10T1/2 cells were obtained from the American Type Culture Collection. 10T1/2 cells were maintained in culture medium (Dulbecco’s modified Eagle’s medium, DMEM) supplemented with 10% of fetal bovine serum (FBS, Thermo Fisher Scientific, # NC0959573) and 1% penicillin/streptomycin (pen/strep, Thermo Fisher Scientific, #15070063). Brown-like adipocyte differentiation was induced by treating confluent 10T1/2 cells with DMEM medium containing 10% FBS, and 1% pen/strep, supplemented with 20 nM insulin (Sigma, #I1507), 1 nM T3 (Sigma, #T2877), 125 μM indomethacin (Sigma, #I7378), 1 μM dexamethasone (Sigma, #D4902), 1 μM rosiglitazone (Sigma, #557366-M), and 0.5 μM isobutylmethylxanthine (Sigma, #I5879). After 48 h of stimulation, the induction medium was removed and a maintenance medium, constituted by DMEM supplemented with 10% FBS, 1% pen/strep, 1 nM T3, and 20 nM insulin, was added and replaced every 2 days.

### Mice

A2AR-FKO mice were generated by crossing Flox-A2AR mice (28) provided by Joel Linden and backcrossed 8 times on a C57BL/6 background with Adiponectin-cre mice (obtained from Jax Labs). Mice were housed under a 12 h light/12 h dark cycle at constant temperature (23 °C), with free access to food and water at the mouse facility of the NYU Medical Center, in New York. The HFD (Research Diet, Cat# D12492) was provided fresh every 2 days and kept at 4 °C for long-term storage.

For cold tolerance tests, 10-week-old control littermates and A2AR-FKO mice were placed at 4 °C for up to 6 h. Measurements of core temperatures were obtained from mice individually caged and exposed to cold with free access to water, using a rectal thermometer (BAT-12, Physitemp), before the start of the experiment and every hour for up to 6 h. At the end of the cold exposure period, mice were euthanized and fat depots harvested for analysis. For HFD studies, 10-week-old male control and A2AR-FKO mice were placed on HFD diet (60% Kcal fat, Research Diet, D124920) for 12 weeks. All animal procedures used in this study were approved by the Institutional Animal Care and Use Committee of NYU Grossman School of Medicine.

### RNA isolation and RT-PCR analysis

Total RNA was obtained from cultured cells using RNeasy (Qiagen, #75144) and from tissues with TRIzol (Thermo Fisher Scientific, #15596018). iScript cDNA Synthesis Kit (BioRad, #1708890) was used to reverse transcribe 1 μg of total RNA into cDNA. RT-PCR analysis was performed using 25 ng of cDNA, 300 nM of primers (listed below) and iQ SYBR Green Supermix (BioRad, #1708880), in triplicate, following the manufacturer’s instructions. We used the ΔΔCt method for relative mRNA quantification by normalizing each sample to the average change in cycle threshold value of the 36B4 gene, which was used as control. The following primers were utilized for Q-PCR analysis: −36B4 Fwd: GCTTCATTGTGGGAGCAGAC; 36B4 Rev: ATGGTGTTCTTGCCCATCAG; A2AR Fwd: GTCCCTACCAAAGCTAGGCTG; A2AR Rev: AAGCACGTTACCCAGGA; A2BR Fwd: CAAGTGGGTGATGAATGTGG; A2BR Rev: TTTCCGGAATCAATTCAAGC; Ucp1 Fwd: GGCCCTTGTAAACAACAAAATAC; Ucp1 Rev: GGCAACAAGAGCTGACAGTAAAT; PGC1α Fwd: ACCATGACTACTGTCAGTCACTC; PGC1α Rev: GTCACAGGAGGCATCTTTGAAG; Dio2 Fwd: AATTATGCCTCGGA GAAGACCG; Dio2 Rev: GGCAGTTGCCTAGTGAAAGGT; Cidea Fwd: TGACATTCATGGGATTGCAGAC; Cidea Rev: CGAGCTGGATGTATGAGGGG; FABP4 Fwd: ATGTGCGACCAGTTTGTG; FABP4 Rev: TTTGCCATCCCACTTCTG; IL-6 Fwd: GACAACTTTGGCATTGTGG; IL-6 Rev: ATGCAGGGATGATGTTCTG; TNFα Fwd: CCAGACCCTCACACTCAGATC; TNFα Rev: CACTTGGTGTGCTACGAC; MCP1 Fwd: AGGTCCCTGTCATGCTTCTG; MCP-1 Rev: GCTGCTGGTGATCCTCTTGT; ZNF638 Fwd: ATTGAGAGCTGTCGGCAGTTA; ZNF638 Rev: GGAATGAGAACGT CTTCTTGGAG; ZFP516 Fwd: AGCGCTTGGATATCCTCAGTA; ZFP516 Rev: GAGGGGCCCTGCTGGCACAGT; PRDM16 Fwd: CCACCAGCGAGGACTTCAC; PRDM16 Rev: GGAGGACTCTCGTAGCTCGAA; HSF1 Fwd: AGGCAGGAGCATAGATGAGA; HSF1 Rev: AGGATGGAGTCAATGAAGG; Cd206 Fwd: CTCTGTTCAGCTATTGGACGC; Cd206 Rev: CGGAATTTCTG GGATTCAGCTTC; Ym1 Fwd: GTCTTGCTCATGTGTGTAAGTGA; Ym1 Rev: CAGGTCTGGCAATTCTTCTGAA.

### Western blot analysis

Whole cell extracts were obtained from cultured cells and from tissues using RIPA buffer containing 20 mM Tris, 150 mM NaCl, 1% NP-40, supplemented with a cocktail of protease inhibitors (Thermo Fisher Scientific, #PIA32953). Twenty micrograms of protein lysates were run on 10% SDS-polyacrylamide gels and transferred on 0.45 μm polyvinylidene fluoride membranes (Millipore, #IPVH00010). Membranes were incubated for 1 h at room temperature with a blocking solution containing 5% nonfat dry milk (w/v) resuspended in TBST 0.1% buffer (50 mM Tris–HCl, 150 mM NaCl, pH 7.4, and 0.1% Tween-20) and subsequently incubated with primary antibodies at 4 °C overnight in a solution containing 1% bovine serum albumin in TBST 0.1% buffer (Thermo Fisher Scientific; #BP9703-100). The following antibodies were used at the dilutions indicated: anti-UCP1 (Abcam, #ab10983), Anti-A2AR (Abcam, cat# ab79714) at 1:1000 and anti-vinculin (Proteintech, #66305-1-Ig) at 1:2000, anti-HSL antibody (CST# 4107) and anti-phospho (CST# 4126) at 1:1000. After antibody incubation, the membranes were washed three times in TBST 0.1% (v/v) and incubated at room temperature for 1 h with a 1:20,000 dilution of anti-rabbit (#1706515) or anti-mouse (#1706516) IgG horseradish peroxidase–conjugated (BioRad) in TBST 0.1% containing 2% nonfat dry milk (w/v). After four additional washes in TBST 0.1% (v/v), immunoblots were developed, using an enhanced chemiluminescence kit (GE Healthcare, #RPN2108) on Hyblot CL autoradiography films (Thomas Scientific #E3012), using an X-ray film developer (Konica Minolta, #SRX-101A).

### Histology

Dissected tissues were fixed in 10% neutral buffered formalin (Fisher) and embedded in paraffin, according to standard procedures. Tissue sections of BAT and scWAT of 5 mm thickness were stained with H&E, and UCP1 immunohistochemistry (Abcam, Cat# ab10983) was performed following manufacturer’s instructions.

### GTT and ITT measurements

GTT and ITT were performed after 10-week-old mice were kept for 12 weeks on HFD. For GTT assays, mice fasted overnight were injected i.p. with glucose (Sigma, cat# G8270) in saline solution (2 g/kg). Measurements of plasma glucose levels in blood obtained from tails were performed at time 0 and subsequently at 15, 30, 60, 90, and 120 min after glucose injection. For ITT analysis, all mice were fasted for 4 h before the test and received an i.p. injection of insulin (Roche, cat# 11061-68-0) resuspended in saline at 1mU/kg. Plasma glucose levels were measured at time 0 and after 15, 30, 60, 90, and 120 min.

### Serum analysis

Serum triglycerides (Sigma, cat# MAK266), cholesterol (Sigma, cat# MAK043), and free fatty acid (Sigma, cat# MAK044) levels were measured as per manufacturer’s instructions in blood obtained from control and A2AR-FKO exposed to HFD for 12 weeks. Leptin concentrations were assessed with mouse Leptin ELISA kit (Ray biotech) and adiponectin levels with mice Adiponectin Elisa kit (Merck Millipore).

### Liver triglyceride analysis

Hundred milligrams of liver samples were homogenized in PBS, and lipids were extracted using chloroform:methanol (2:1) and 0.1% sulfuric acid. The organic phase was collected, dried, and resuspended in isopropanol. The levels of triglycerides in liver samples were determined by using the reagents provided in the triglyceride quantification kit (Sigma, cat# MAK266), following the manufacturer’s instructions, and normalized for liver weights.

### Oxygen consumption analysis and mitochondrial DNA measurements

10-week-old littermate mice of the two genotypes were placed in metabolic cages (Columbus Instruments) at 22 °C, with 50% humidity, 12 h light/dark cycle, with light on at 6:30 AM and off at 6:30 PM, for 3 days to acclimate; subsequently O2, CO2, food and water intake, and locomotor activity were measured while mice were fed a chow diet for 3 days. All the metabolic studies were performed by the Rodent Behavior Laboratory at the NYU Grossman School of Medicine. Cellular metabolic rates and mitochondrial DNA (mtDNA) measurements were performed in differentiated adipocytes derived from stromal vascular fraction (SVF) cells obtained from the scWAT of control and A2AR-FKO mice. Respiration was measured under basal conditions, following the addition of ATP synthase inhibitor oligomycin, the mitochondrial uncoupler FCCP, or the complex III inhibitor antimycin A using a XF24 Analyzer (Agilent Biosciences). mtDNA was measured as the ratio between the cytochrome c oxidase subunit I gene of the mtDNA and the NDUFV1 nDNA gene. The primers used are the following: for cytochrome c oxidase subunit I, 5-TGC TAG CCG CAG GCA TTA C-3 (forward primer) and 5-GGG TGC CCA AAG AAT CAG AAC-3 (reverse primer); for NDUFV1, 5-CTT CCC CAC TGG CCTCAA G-3 (forward primer) and 5-CCA AAA CCC AGT GAT CCA GC-3 (reverse primer) (29). For PCR sample preparation, 5 μl of genomic DNA (40 ng/ml) was mixed with 1 μl of each primer (10 μM), 3 μl of nuclease-free water, and 10 μl of SYBG master enzyme mix. The reaction was initiated at 94 °C for 10 min, followed by 40 cycles through 94 °C 10 s, 60 °C 30 s, and 94 °C 10 s. All reactions were run in triplicate.

### Primary cell isolation and lipolysis

Primary cells were isolated from the scWAT of control and A2AR-FKO animals using Collagenase/Dispase (Cat # 11097113001, Roche). Briefly, tissues were washed twice with the PBS +1% P/S solution, minced, and incubated for 45 min in a 37 °C incubator in a shaking mode. Next, DMEM medium containing 10% of FBS was added to the cells to neutralize the collagenase activity, mixed, and spun at 1200*g* for 4 min. Upper layer containing mature adipocytes was removed and pellets were resuspended in 3 ml of DMEM growth media. For lipolysis assays, SVF cells were differentiated into brown-like adipocytes in a 12-well plate for 4 days. For the stimulation of lipolysis, differentiated cells were treated with 10 μM isoproterenol for 30 min and cells were harvested for protein extraction using 1X RIPA buffer, as described in Western blot section.

### Glucose uptake colorimetric assay

For glucose uptake assays, SVF cells were differentiated for 5 days and measurements were performed using the Glucose Uptake Colorimetric Assay Kit (Sigma-Aldrich, cat # MAK08).

### Flow cytometry analysis

WAT was collected from the peritoneal cavity of control or A2AR-FKO male mice, enzymatically digested with type II collagenase (1 mg/ml) and porcine pancreatic elastase (0.1 mg/ml) at 37 °C for 45 min and filtered through a 70 μm strainer to generate a single cell suspension. Samples were stained for 30 min in 2% bovine serum albumin with Zombie NIR, anti-CD45, anti-Cd11b, anti-Cd11c, anti-Ly-6g, anti-Ly-6c, anti-F4/80, anti-CD206, anti-CD68, anti-Lyve1, anti-CD3, anti-CD19, anti-CD4, anti-CD8, anti-Nkp46, and processed on an Attune N x T flow cytometer. Data were acquired on Attune software (flow-cytometers/attune-nxt-flow-cytometer/software.html) and analysis performed in FlowJo software (FlowJo Tm v10 software).

### Statistical analysis

The results obtained are expressed as mean ± SD, unless otherwise noted. Student’s *t* test or one-way ANOVA was used for comparison between groups. *p* values < 0.05 were considered statistically significant. The statistical analyses were performed using the Prism 7 software (GraphPad Software).

## Data availability

The data underlying this article are available in the article. Additional data underlying this article will be shared on reasonable request to the corresponding author.

## Supporting information

This article contains supporting information.

## Conflict of interest

B. N. C. owns stocks in Regenosine. All authors declare that they have no conflicts of interest with the contents of this article.

## Figures and Tables

**Figure S3:**