# Supplementary material for: Metabolic dysfunction in mice with adipocyte-specific ablation of the adenosine A2A receptor
Source: J Biol Chem. 2025 Jan 17;301(2):108206. doi: 10.1016/j.jbc.2025.108206 (PMC11850162; doi:10.1016/j.jbc.2025.108206)
Supplement: Supporting information [file mmc1.docx]

**Supporting information**

**Figure S1: Weight of control and A2AR-FKO mice on a ND and levels of adenosine receptors in adipose tissue.** (**A**) Weight of BAT, scWAT and eWAT of control and A2AR-FKO mice on a normal diet. (**B**) Relative mRNA levels of A2AR and A2BR in scWAT of control and A2AR-FKO mice. (n=3).

**Figure S2: Gating strategy**. Gating strategy used for the analysis of eWAT obtained from control and A2AR-FKO mice treated for 12 weeks with a HFD.

**Figure S3: Sea horse data analysis**. Quantification of basal respiration and proton leak data in brown differentiated adipocytes obtained from SVF cells obtained from scWAT of control and A2AR-FKO mice.
